# The Epstein-Barr Virus G-Protein-Coupled Receptor Contributes to Immune Evasion by Targeting MHC Class I Molecules for Degradation

**DOI:** 10.1371/journal.ppat.1000255

**Published:** 2009-01-02

**Authors:** Jianmin Zuo, Andrew Currin, Bryan D. Griffin, Claire Shannon-Lowe, Wendy A. Thomas, Maaike E. Ressing, Emmanuel J. H. J. Wiertz, Martin Rowe

**Affiliations:** 1 Cancer Research-UK Institute for Cancer Studies, School of Cancer Sciences, University of Birmingham, Edgbaston, Birmingham, United Kingdom; 2 Center of Infectious Diseases and Department of Medical Microbiology, Leiden University Medical Center, Leiden, The Netherlands; 3 Department of Medical Microbiology, University Medical Centre Utrecht, Utrecht, The Netherlands; Oregon Health & Science University, United States of America

## Abstract

Epstein-Barr virus (EBV) is a human herpesvirus that persists as a largely subclinical infection in the vast majority of adults worldwide. Recent evidence indicates that an important component of the persistence strategy involves active interference with the MHC class I antigen processing pathway during the lytic replication cycle. We have now identified a novel role for the lytic cycle gene, *BILF1*, which encodes a glycoprotein with the properties of a constitutive signaling G-protein-coupled receptor (GPCR). BILF1 reduced the levels of MHC class I at the cell surface and inhibited CD8^+^ T cell recognition of endogenous target antigens. The underlying mechanism involves physical association of BILF1 with MHC class I molecules, an increased turnover from the cell surface, and enhanced degradation via lysosomal proteases. The BILF1 protein of the closely related CeHV15 γ_1_-herpesvirus of the Rhesus Old World primate (80% amino acid sequence identity) downregulated surface MHC class I similarly to EBV BILF1. Amongst the human herpesviruses, the GPCR encoded by the ORF74 of the KSHV γ_2_-herpesvirus is most closely related to EBV BILF1 (15% amino acid sequence identity) but did not affect levels of surface MHC class I. An engineered mutant of BILF1 that was unable to activate G protein signaling pathways retained the ability to downregulate MHC class I, indicating that the immune-modulating and GPCR-signaling properties are two distinct functions of BILF1. These findings extend our understanding of the normal biology of an important human pathogen. The discovery of a third EBV lytic cycle gene that cooperates to interfere with MHC class I antigen processing underscores the importance of the need for EBV to be able to evade CD8^+^ T cell responses during the lytic replication cycle, at a time when such a large number of potential viral targets are expressed.

## Author Summary

Epstein-Barr virus (EBV) is a herpesvirus and an important human pathogen that can cause diseases ranging from non-malignant proliferative disease to fully malignant cancers of lymphocytes and epithelial cells. Nevertheless, the vast majority of people in all populations worldwide are infected with EBV. After primary infection the virus persists for the life of the infected individual, and usually without clinical symptoms. To understand how EBV causes disease, we also need to know how this virus is able to persist in healthy individuals with potent immune-responses. A picture is emerging which suggests that EBV, similar to non-tumorigenic herpesvirus, has multiple co-operating mechanisms to modulate immune responses by interfering with antigen processing pathway in cells undergoing lytic virus replication. We have now identified an EBV-encoded protein, BILF1, which targets MHC class I molecules for lysosomal degradation, leading to impaired recognition by immune T cells. BILF1 is a constitutively active G-protein-coupled receptor, but its effect on MHC class I degradation is independent of its signaling functions, and the molecular mechanisms are distinct from those identified in other viruses that induce degradation of MHC class I. This work aids our understanding of EBV biology and emphasizes the complexity of mechanisms evolved by viruses to enable persistent infection.

## Introduction

Viruses that persist asymptomatically in infected individuals need to evade or modulate immune responses, and particularly the adaptive T cell responses. A universal mechanism for virus persistence involves latency, where viral genes are transcriptionally silenced and, therefore, not available as targets for immune effectors. In addition, viruses may exhibit active immune-evasion mechanisms. In this context, the herpesviruses are paradigmatic. For example, the human cytomegalovirus (HCMV, a β-herpesvirus), encodes at least 25 proteins with immune-modulating functions [1], of which 5 can impair antigen presentation via MHC-class I to the CD8^+^ T cell responses: US6 binds to the transporter associated with antigen processing (TAP) complex to inhibit peptide transport from the cytosol to the ER [2]–[4], US3 [5]–[7] and US10 [8] interfere with the maturation of MHC class I heavy chains and their egress from the ER, while US2 and US11 trigger retrograde transport of MHC class I from the ER to the cytosol with subsequent proteasome-mediated degradation [9]–[11].

Epstein-Barr virus (EBV) is a γ_1_-herpesvirus whose normal biology is fundamentally distinct from most other human herpesviruses in that it has growth-transforming activity for human B cells [12]. This provides an alternative strategy for replicating virus genomes in proliferating cells without necessarily producing infectious viral progeny. This is a potentially dangerous strategy and it is unsurprising, therefore, that EBV is associated with the pathogenesis of certain human cancers [12],[13]. Nevertheless, EBV has evolved to persist asymptomatically in most immunocompetent individuals, and it is ubiquitous in adult populations worldwide. This successful persistence is largely attributable to the ability of EBV to establish various types of ‘latency’ (i.e. not producing infectious virus progeny) in lymphoid cells [14]. It is notable that the most restricted forms of latency (which involve expression either of no viral genes, or of non-coding small RNAs and possibly the poorly immunogenic EBNA1 and/or LMP2A proteins) are normally associated with quiescent cells and can evade recognition by CD8^+^ T cells [12],[14],[15]. In contrast, the form of latency associated with growth transformation is characterized by expression of at least 8 viral proteins, including immunodominant antigens [12],[14],[15], together with enhanced expression of antigen presentation components [16],[17] that render the cells susceptible to recognition and elimination by EBV-specific CD8^+^ cytotoxic T cell responses *in vivo* [12],[14],[15]. The increased incidence of EBV-associated B lymphoproliferative disease in iatrogenically immunosuppressed transplant patients [12],[18] emphasizes the need for adaptive T cell immune responses to prevent uncontrolled proliferation of EBV-transformed B cells.

While the interaction of EBV with the host immune responses differs from non-oncogenic herpesviruses during the growth-transforming phase of virus infection, it does share many properties with non-oncogenic herpesviruses during the lytic virus replication phase of the viral life-cycle. Responses to EBV lytic cycle antigens dominate the EBV-specific T cell response in both primary and persistent infection with the virus, but the ability of CD8^+^ T cells to recognize EBV-infected B cells in lytic cycle is compromised by a reduced expression of cell surface MHC class I [15],[19],[20]. The levels of cell surface MHC class I in lytic cycle reflect active interference with the antigen processing pathway by mechanisms that are poorly understood but which are at least in part due to an impairment of TAP-dependent peptide transport into the ER [21] and by host protein synthesis shutoff [22]. The early lytic cycle genes, *BNLF2a* and *BGLF5* were recently identified as genes targeting TAP function [23] and host-shutoff [22],[24] respectively. While BGLF5 protein remains expressed throughout lytic cycle, BNLF2a protein expression decreases within 24 hr of induction and probably only acts during the early stages of lytic cycle (N. Croft, D. Horst et al, manuscript in preparation).

In the present study, we have identified a third lytic cycle gene that actively interferes with MHC class I antigen presentation to CD8^+^ T cells by increasing the turnover of MHC class I molecules at the cell surface and targeting them for lysosomal degradation. This new immune-evasion function of EBV mapped to *BILF1*, which was previously identified as a rhodopsin-like seven-transmembrane segment G-protein coupled receptor (GPCR) with constitutive signaling functions [25]–[27]. We have characterized the molecular mechanisms by which BILF1 increases MHC class I turnover, and found them to be independent of its G-protein activating function and distinct from the mechanisms employed by other herpesviruses that target MHC class I for degradation.

## Results

### BILF1 affects MHC class I expression and antigen presentation to CD8^+^ T cells

**Figure 1:**
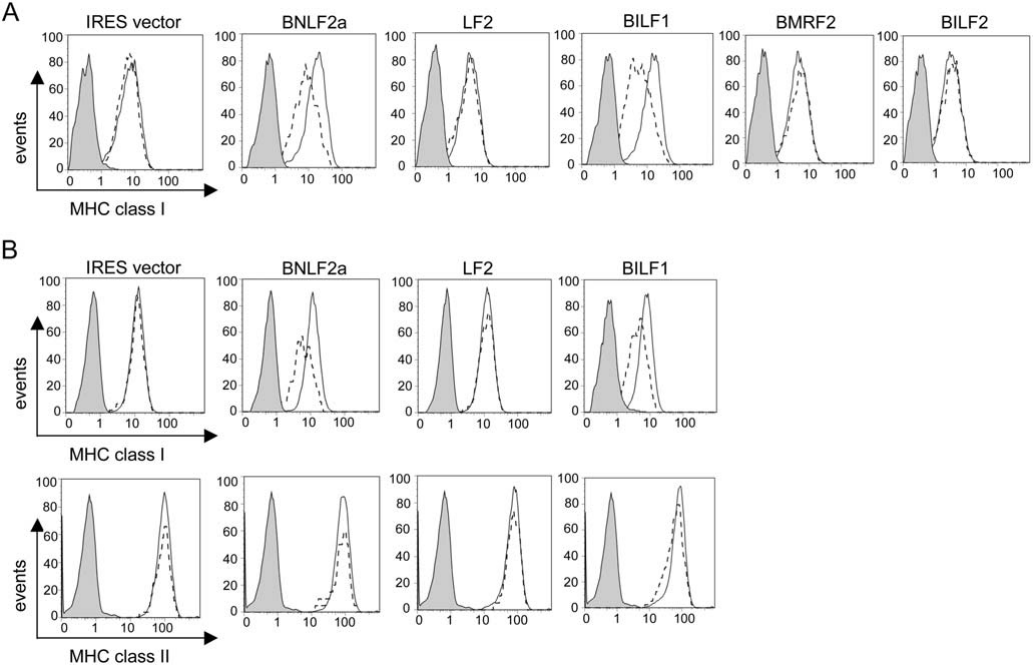
*BILF1* identified as a lytic gene that downregulates surface MHC class I. 293 (A) or MJS (B) cells were transfected with different EBV genes in the bi-cistronic vector, pCDNA3-IRES-nlsGFP. At 48 hr post-transfection, surface MHC class I was stained with PE-conjugated W6/32 mAb and (in MJS only) MHC class II was stained with PE-conjugated anti-DR mAb, YE2/36-HLK. Two-colour flow cytometry was used to analyse staining in the untransfected GFP^−^ population, shown as the solid line histogram, and in the transfected GFP^+^ population, shown as the dashed line histogram. The grey histogram denotes background staining obtained with an isotype control PE-conjugated antibody.

We initiated a systematic screen of the EBV lytic genes to identify viral products that might affect MHC class I expression. EBV genes were cloned into the bicistronic vector pCDNA3-IRES-nls-GFP, which co-expresses the inserted gene with GFP, then transfected into 293 and MJS cells. At 48 h after transfection, the cells were stained with PE-conjugated W6/32 mAb and the levels of surface MHC class I molecules were measured by flow cytometry. Representative results of W6/32 staining in transfected 293 cells are shown in Fig. 1A. As a negative control, ‘empty’ pCDNA3-IRES-nls-GFP vector was transfected, and as a positive control, a known immune-evasion gene was transfected using the pCDNA3-BNLF2a-IRES-nls-GFP vector. The results show that the *LF2*, *BMRF2*, and *BILF2* had no effect on MHC class I levels, whereas *BILF1* caused a reduction comparable to *BNLF2a*. In addition, two other EBV genes, *LF1* and *BXLF2* were also screened in the same set of experiments and had no effect on MHC class I levels (data not shown). This assay was then extended to a second cell line (MJS) chosen for its expression of MHC class II as well as MHC class I molecules, which confirmed the downregulation of MHC class I by *BILF1*, with no effect on the levels of MHC class II (Fig. 1B).

**Figure 2:**
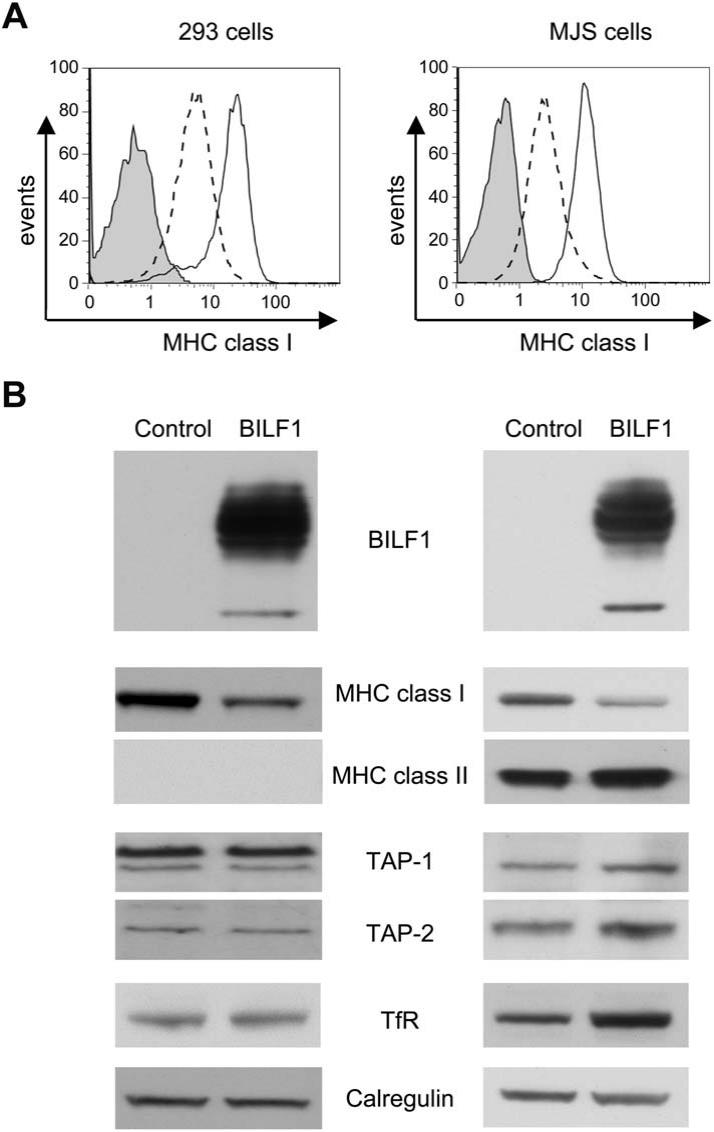
Characterization of cells stably transduced with a BILF1 retroviral vector. (A) 293 or MJS cells were stably transduced with control (pQCXIH) or BILF1 (pQCXIH-HABILF1) retrovirus. Surface MHC class I molecules were stained with PE-conjugated W6/32 antibodies and analyzed by flow cytometry. The solid line histograms depict the surface HLA class I staining of control cell lines, while the dashed line histogram depicts the surface HLA class I staining of cell lines expressing BILF1. The grey histogram illustrates background staining obtained with an isotype control PE-conjugated antibody. (B) Total cell lysates were generated from the retrovirus-transduced 293 and MJS cell lines, and 2×10^5^ cell equivalents were separated by SDS-PAGE and analyzed by Western Blotting with mAbs specific for BILF1 (3F10, anti-HA tag), MHC class I (HC10), MHC class II (DA6.147), TAP-1 (148.3), TAP-2 (435.3), TfR (H68.4) or with polyclonal antibodies to calregulin as a loading control.

These screening experiments suggested a specific effect on surface MHC class I expression by BILF1. To examine this in more detail, we generated a retroviral expression vector for BILF1, and transduced both 293 and MJS cells to generate stable cell lines expressing BILF1. Since the BILF1 in these retroviral vectors contained an N-terminal HA-tag sequence, expression of BILF1 in the transduced cells was initially confirmed by staining of viable cells with anti-HA mAb and flow cytometry analysis (data not shown). Staining with PE-W6/32 mAb confirmed that expression of MHC class I expression at the cell surface was reduced in BILF1-expressing 293 and MJS cells relative to paired lines transduced with a control retrovirus vector (Fig. 2A). This effect was reproducibly stronger in the stable retroviral transduced cells than in the previous transient-transfection experiments. No downregulation of MHC class II in MJS, nor of transferrin receptor (TfR) in 293 or MJS, was observed by flow cytometry (data not shown). Western blots of whole cell lysates showed that the effect of BILF1 on the levels of cell surface MHC class I were reflected by a similar decrease in the amount of total cellular MHC class I heavy chains (Fig. 2B). Notably, the levels of TAP-1 and TAP-2 components of the peptide transporter complex and calregulin were unaffected by expression of BILF1 (Fig. 2B). Levels of TfR receptor were unaffected in 293 cells but reproducibly showed a small increase, along with MHC class II, in MJS cells (Fig. 2B).

**Figure 3 ppat-1000255-g003:**
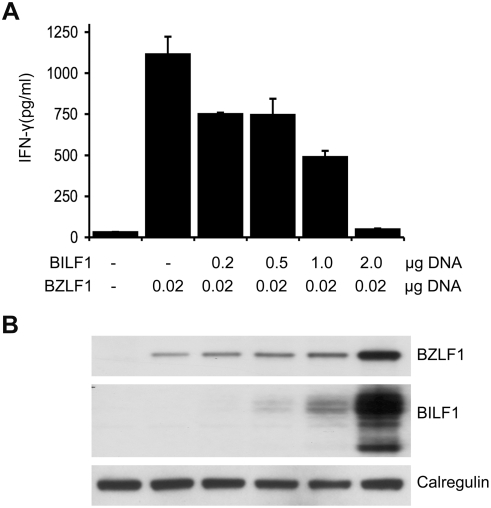
BILF1 inhibits T cell recognition of endogenous EBV antigen in MJS cells. (A) MJS cells were co-transfected with 0.02 µg p509 plasmid (BZLF1 expression vector) and different amounts (0–2 µg) of pCDNA3-HABILF1-IRES-nlsGFP bulked to a constant amount of DNA with control plasmid. At 24 hr post-transfection, the MJS cells were co-cultured with CD8^+^ effector ‘RAK’ T cells for a further 18 hrs and the supernatants were tested for the release of IFN-γ as a measure of T cell recognition. All results are expressed as IFN-γ release in pg/ml and error bars indicate standard deviation of triplicate cultures. (B) Total cell lysates were generated from the above transfections, and 2×10^5^ cell equivalents were separated and analyzed by Western Blotting using antibodies specific for BZLF1, HA tag (BILF1), or calregulin as a loading control.

The aforementioned results raised the possibility that BILF1 might cause an impairment of the antigen processing pathway that would affect antigen recognition by CD8^+^ T cell responses. To test this hypothesis, HLA-B8 positive MJS cells were transiently transfected with p509 plasmid together with control pCDNA3-IRES-nlsGFP vector or different amounts of pCDNA3-BILF1-IRES-nlsGFP. The p509 vector expresses BZLF1, an EBV lytic cycle protein that is the target of the HLA-B8 restricted ‘RAK’ CD8^+^ T cell effector clone. Following co-culture of RAK T cells with the transfected MJS target cells, the release of IFN-γ was assayed by ELISA as a measure of T cell recognition. The representative experiment in Fig. 3A shows that the RAK clone did not respond to vector control transfected MJS, but showed clear recognition of cells transfected with *BZLF1*. This recognition was inhibited in a dose-dependent manner by co-transfection of *BILF1*, exceeding 90% inhibition at the highest dose of pCDNA3-BILF1-IRES-nlsGFP transfected. Similar results were obtained in 3 separate experiments. Western-blots of the transfected target cells (.. 3B) showed that BILF1 protein expression increased with increasing amounts of pCDNA3-BILF1-IRES-nlsGFP plasmid, as expected. Interestingly, the amount of BZLF1 protein also showed a smaller but reproducible concomitant increase in expression, possibly as a result of constitutive BILF1 signaling (e.g. activation of NFκB; see below) enhancing the CMV promoter driving *BZLF1* expression in p509. A similar inhibition of EBV-specific CD8^+^ T cell recognition by BILF1 was observed in additional experiments performed with 293 cells transfected with the BMLF1 target recognized by HLA-A2 restricted T cell clones (data not shown).

**Figure 4:**
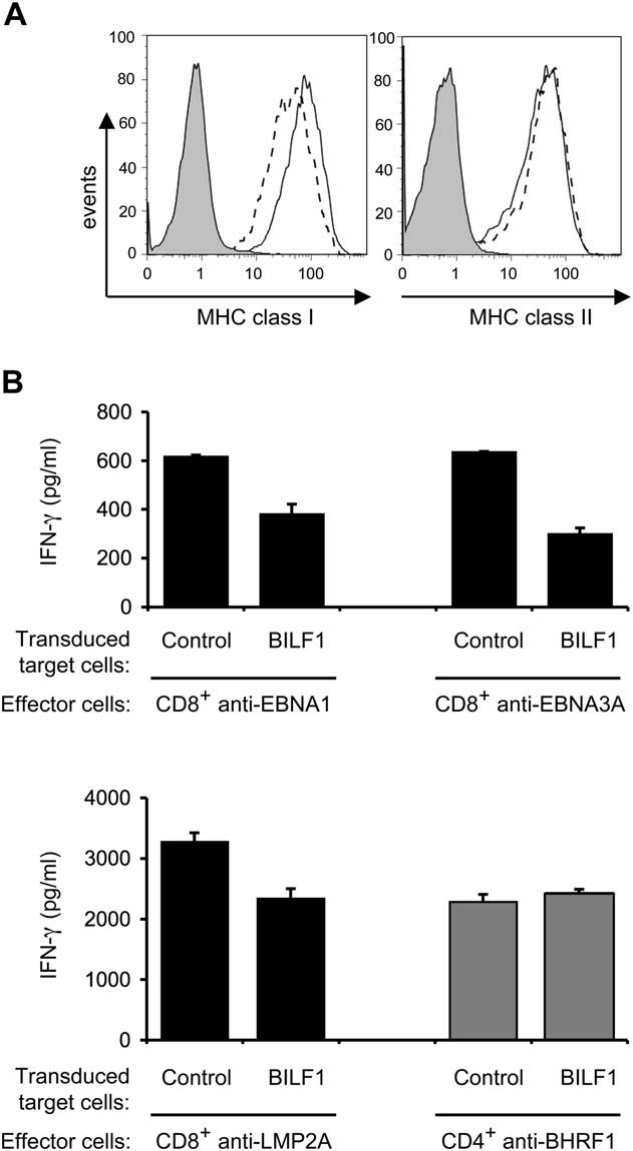
BILF1 downregulates surface MHC class I expression and inhibits the T cell recognition of endogenous EBV antigen in LCLs. (A) LCLs were transduced with PLZRS-HABILF1-IRES-GFP retrovirus. After 6 days, surface MHC class I was stained with PE-conjugated W6/32 mAb and MHC class II was stained with PE-conjugated anti-DR mAb, YE2/36-HLK. Two-colour flow cytometry was used to analyze staining in the untransduced, GFP^−^, population, shown as the solid line histogram, and in the transduced GFP^+^ (BILF1^+^) population, shown as the dashed line histogram. The grey histogram denotes background staining obtained with an isotype control PE-conjugated antibody. (B) LCL cultures transduced with control retrovirus or with the BILF1 retrovirus were sorted by flow cytometry to generate GFP^+^/BILF1^−^ and GFP^+^/BILF1^+^ lines to use as targets in assays with EBV-specific T cells. The control and BILF1^+^ LCL targets were incubated with HLA-matched CD8^+^ effector T cells clones specific for EBNA1 (HPV), EBNA3A (YPL), or LMP2A (CLG) peptides, or a CD4^+^ effector T cell clone specific for a BHRF1 (PYY) peptide. After 18 hrs the supernatants were tested for the release of IFN-γ as a measure of T cell recognition. All results are expressed as IFN-γ release in pg/ml and error bars indicate standard deviation of triplicate cultures.

In healthy infected individuals, EBV latency is established in the B lymphocyte pool, which implies that at some stage lytic cycle must occur in B cells for the virus to be transmitted to epithelial cells or other hosts. We therefore examined the effect of BILF1 expression in B cells. To simplify the experiment, we chose to use LCLs that had been established by infection of normal resting B cells with a BZLF1 knock-out recombinant EBV; this provided us with a range of endogenous latent proteins as target antigens in a good antigen-presenting host cell which was unable to enter lytic cycle and, therefore, unable to express BILF1 from the viral genome. These LCL were transduced with a PLZRS-BILF1-IRES-GFP retrovirus, and 6 days later were stained for surface MHC class I and class II molecules with PE-conjugated antibodies. The GFP positive (BILF1^+^) population was shown by flow cytometry to express almost 40% less surface MHC class I compared to the GFP negative (BILF1^−^) population in the same culture (Fig. 4A). The expression of BILF1 did not affect the expression of surface MHC class II molecules. The reduction in the expression of surface MHC class I by BILF1 was smaller than that seen in 293 and MJS cells (Fig. 2), but was reproducibly observed in 3 experiments with two different LCLs.

To examine the functional significance of this BILF1-mediated reduction of surface MHC class I expression, EBV-specific T cell recognition experiments were performed on GFP^+^ cells isolated on a fluorescence activated cell sorter after transduction with either a control retrovirus (PLZRS-IRES-GFP) or the BILF1 retrovirus (PLZRS-BILF1-IRES-GFP). These cells were used as targets in T cell assays with different effector cell clones specific for constitutively-expressed latent EBV proteins. The representative experiments in Fig. 4B show that CD8^+^ effectors specific for one of three different latent proteins (EBNA1, HLA-B35 restricted; EBNA3A, HLA-B35 restricted; and LMP2A, HLA-A2 restricted) all recognized the BILF1-expressing LCLs less efficiently than the control LCLs. In contrast, recognition of BHRF1 antigen by HLA-DR4 restricted CD4^+^ effectors was not significantly different between the control and BILF1-expressing LCLs (Fig. 4B). Similar results were obtained in 3 separate experiments.

### BILF1 decreases the half-life of newly-synthesised MHC class I molecules

To further examine the mechanism of the effect of BILF1 on MHC class I, we conducted a pulse-chase analysis of radio-labeled molecules to study the fate of newly synthesized MHC class I molecules. Control and BILF1^+^ 293 cells were pulse-labeled with ^35^S-methionine/cysteine and chased at 37°C in medium containing cold methionine and cysteine. At each time point, cells were lysed in NP40 detergent buffer and immunoprecipitated with mAb W6/32, which reacts with properly assembled MHC class I complexes.

**Figure 5:**
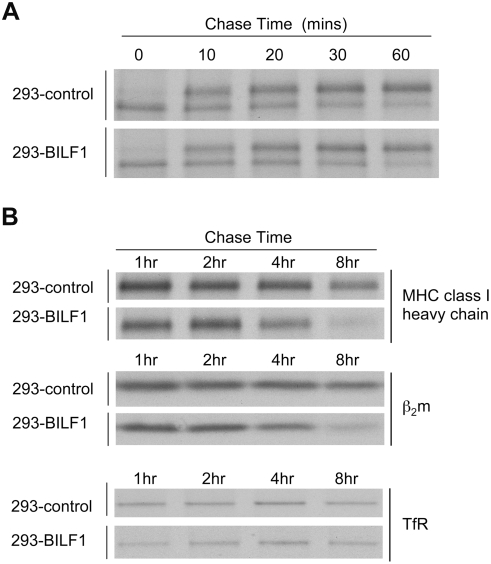
Effect of BILF1 on maturation and degradation of MHC class I molecules. (A) Acquisition of endoglycosidase H (Endo H) -resistance of MHC class I heavy chain. 293 cells (2×10^6^) stably transduced with control or BILF1 retrovirus were metabolically labeled for 15 min with ^35^S-methionine/cysteine and chased for the indicated time periods. After lysis in NP-40 detergent buffer, samples were immunoprecipitated with mAb W6/32 and treated with Endo H enzyme. Protein samples were separated by 10% acrylamide SDS/PAGE gel, dried and exposed to autoradiography. (B) Kinetics of MHC class I molecule degradation. 293 cells (2×10^6^) stably transduced with control or BILF1 retrovirus were metabolically labeled for 15 min and chased for the indicated time periods. After lysis in NP-40 detergent buffer, samples were immunoprecipitated with mAb W6/32 (HLA class I heavy chain and β_2_-microglobulin) or H68.4 (TfR). Protein samples were separated by 10% SDS/PAGE gel, dried and exposed to autoradiography.

In the first set of pulse-chase experiments, the immunopreciptated MHC class I heavy chains were treated with endoglycosidase H (Endo-H), an enzyme which deglycosylates newly-synthesized heavy chains but not the mature form that has been exported from the ER. Most MHC class I heavy chains were Endo-H-sensitive at the beginning of the chase, as expected for newly synthesised heavy chain in the ER. Within 30–60 min, nearly all heavy chains became Endo-H-resistant. The rate of conversion of MHC class I heavy chain to the Endo-H resistant form was similar in control 293 cells and in BILF1^+^ 293 cells (Fig. 5A). These results indicate that the export of MHC class I from the ER and its passage through the Golgi is not impeded by BILF1, and that BILF1 most probably acts at a later stage.

In order to compare the half life of MHC class I molecules, cells were pulsed with radiolabel for 15 min, then chased for up to 8 h before lysis and immunoprecipitation. As shown in Fig. 5B (upper and middle panels), both MHC class I heavy chain and its associated β2-microglobulin showed a half-life of around 8 h in the control 293 cells, which was reduced to around 5 h in BILF1^+^ 293 cells. In the same experiment, immunoprecipitations of the TfR showed no difference between control and BILF1^+^ 293 cells (Fig. 5B, lower panel).

It is worth noting that over a series of experiments the incorporation of radiolabel into MHC class I molecules was not reduced in BILF1-expressing 293 cells relative to the control cells, indicating that BILF1 did not inhibit their translation.

### Physical association of BILF1 with MHC class I complexes

**Figure 6:**
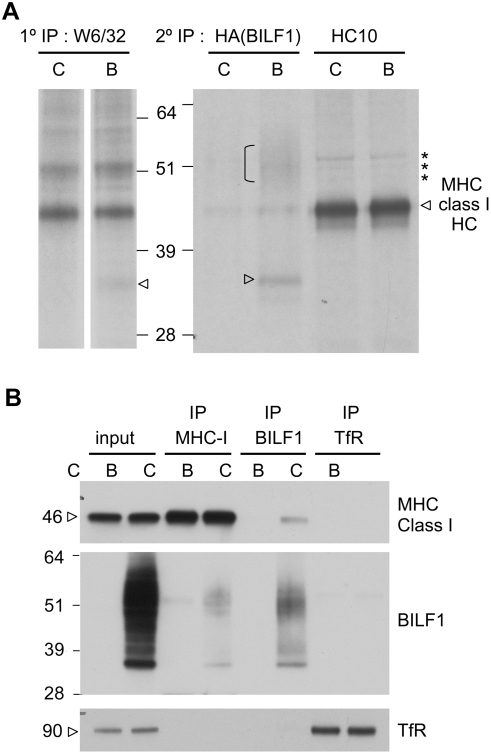
BILF1 is physically associated with the MHC class molecule complex. (A) 293 cells (10^7^) stably transduced with control (c) or BILF1 (b) retrovirus were metabolically labeled for 15 min and chased for 20 min. After lysis in NP-40 buffer and immunoprecipitation with mAb W6/32, the samples were dissociated by boiling in reducing sample buffer, and were re-precipitated with either 3F10 ( HA tag on BILF1) or HC10 (MHC class I heavy chain). Protein samples were separated by 10% acrylamide SDS/PAGE gel, dried and exposed to autoradiography. The arrowhead and bracket indicate the presence of 33 kD and 45–55 kD BILF1 bands, whilst the asterisks indicate probable ubiquitinated MHC class I species. (B) 293 cells (2×10^6^) stably transduced with control (c) or BILF1(b) retrovirus were treated with concanamycin A (50 nM) for 20 hr prior to lysing the cells with NP40 detergent buffer and immunoprecipitation with antibodies specific for MHC class I (W6/32), HA tagged BILF1 (12CA5), or TfR (H68.4). Cell lysates and immune complexes were separated by SDS/PAGE gel, and analyzed by western blotting using antibodies specific for MHC class I (HC10), HA tagged BILF1 (3F10), and TfR (H68.4). The first two samples on the gel are total cell lysates representing 5% of the input lysate for immunoprecipitations.

In the pulse-chase and immunoprecipitation of MHC class I, an additional co-precipitating band at around 33 kD was reproducibly observed in W6/32 immunoprecipitates of BILF1-293 lysates but not in control lysates (data not shown). To test the hypothesis that this band might be the smaller, non-glycosylated, form of BILF1 co-precipitating with MHC class I, we carried out a second round of precipitations on eluates from the W6/32 precipitations. The eluates were first diluted in lysis buffer, then equal aliquots were re-precipitated either with a mAb to the HA-tag of BILF1 or with the HC10 mAb to MHC class I free heavy chain. As shown in Fig. 6A, re-precipitation of the W6/32 eluate from BILF1^+^ 293 cells with anti-HA revealed a band at around 33 kD and a smear at 48–55 kD (lane 2) that were not present in the re-precipitation with HC10 mAb (lane 4), and neither were they detectable in the anti-HA re-preciptate of the control 293 sample (lane 1). These experiments indicated that BILF1 associated with the MHC class I complex. It is also worth noting that re-precipitation with HC10 mAb pulled down a ladder of bands above the MHC class I heavy chain (indicated with asterisks in Fig. 6A), which probably represent ubiquitinated MHC molecules; the intensity of these bands was indistinguishable between control 293 and BILF1^+^ 293 cells.

To confirm the association of MHC class I with BILF1, additional immunoprecipitations were conducted with lysates from non-radiolabeled cells. Here, control 293 and BILF1^+^ 293 cells were again solubilized in NP40 detergent buffer, then immunoprecipitated with W6/32 (MHC class I), anti-HA (BILF1), or anti-TfR, before analysis by Western blotting. As shown in Fig. 6B, some MHC class I molecules were co-precipitated by anti-HA mAb (lane 6, top blot), and some BILF1 molecules were co-precipitated by W6/32 mAb (lane 4, middle blot). In the control TfR immunoprecipitations, no co-precipitation of MHC class I or BILF1 was observed (lane 8, top and middle blots). Taken together, the radiolabeled and cold immunoprecipitation data provide compelling evidence that BILF1 physically associates with the MHC class I molecules. Indeed, since weak co-precipitating BILF1 bands were even seen immediately following a 15 min pulse-label (data not shown), it is likely that the interaction first occurs in the ER.

### BILF1 is associated with MHC class I molecules on the cell surface and increases their rate of internalization

For other viral proteins known to target MHC class I molecules for degradation, three broad mechanisms have been identified: either they induce retrograde transport from the ER to the cytosol for degradation by proteasomes, as exemplified by HCMV US2 and US11 [9]–[11]; or they redirect them to endolysosomal vesicles for degradation, exemplified by gp48 of murine CMV [28]; or they trigger enhanced endocytosis of MHC class I from the cell surface, followed by lysosomal degradation, as exemplified by K3 and K5 of KSHV [29].

**Figure 7:**
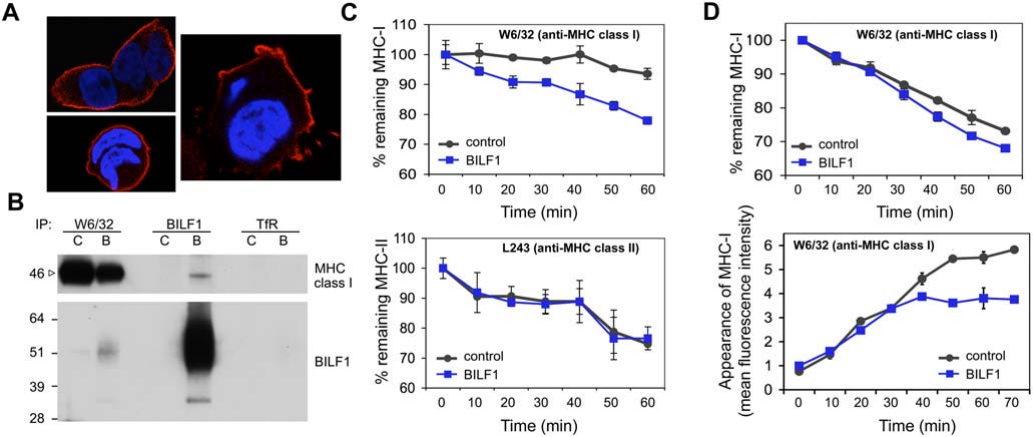
BILF1 associates with MHC class I molecules at the cell surface and increases their rate of internalization. (A) BILF1 is predominantly localized at the cell surface. 293 cells stably transduced with BILF1 retrovirus were grown on glass slides, fixed and permeabilized, then stained with rat anti-HA (3F10) primary antibodies and Alexa Fluor® 594 goat anti-rat IgG. The nuclei were counterstained with DAPI. The stained slides were analyzed with a laser scanning confocal microscope, and the three photographs show different 1 micron-thick sections through representative cells. BILF1 stained red, and the nuclei stained blue. (B) BILF1 and MHC class I molecules co-precipitate at the cell surface. 293 cells (2×10^6^) stably transduced with control (c) or BILF1 (b) retrovirus were incubated with saturating concentrations of antibodies specific for MHC class I (W6/32), TfR (H68.4) or HA tagged BILF1 (3F10) on ice. After washing away excess antibody, the cells were lysed with NP40 detergent buffer, then precipitated with protein A/G beads and subjected to western-blotting as in Fig. 6B, using antibodies specific for MHC class I (HC10) and HA tagged BILF1 (3F10). (C) BILF1 increases the rate of internalization of MHC class I, but not class II, from the cell surface. MJS cells stably transduced with control or BILF1 retrovirus were incubated at 0°C with saturating concentrations of mAb to MHC class I (W6/32; top graph) or MHC class II (L234; bottom graph), then washed and incubated at 37°C for different periods of time. The cells were subsequently stained with PE-conjugated goat anti-mouse IgG antibody, and analyzed by flow cytometry. The mean fluorescence intensities of staining were averaged for triplicate samples, and normalized to the initial time 0 min samples. (D) BILF1 increases the rate of internalization, but not the rate of appearance, of MHC class I at the cell surface. Top graph: 293 cells stably transduced with control or BILF1 retrovirus were incubated at 0°C with saturating concentrations of mAb to MHC class I (W6/32), then treated exactly as for the internalization assay performed with MJS cells in panel C. Bottom graph: replicate aliquots of the saturated W6/32-bound cells were harvested at the indicated time points, and the appearance of new MHC class I molecules was assayed by staining with PE-conjugated W6/32 antibody. The mean fluorescence intensities of staining were averaged for triplicate samples.

In order to investigate which of these mechanisms might be employed by BILF1, we first examined the cellular localisation of BILF1. Confocal microscopy of BILF1-espressing 293 cells showed that BILF1 was predominantly located in the plasma membrane (Fig. 7A). Since antibodies to the HA-tag engineered to the N-terminus of BILF1 allowed detection of BILF1 at the surface of viable cells, we were able to examine whether the association between MHC class I molecules and BILF1 can also be demonstrated at the cell surface. Thus, viable control 293 and BILF1^+^ 293 cells were first incubated with W6/32 (MHC class I), anti-HA (BILF1), or anti-TfR on ice to allow the antibodies to bind to the surface antigen. After removal of excess unbound antibodies by washing, the cells were then solubilized in NP40 detergent buffer and the antibody∶antigen complexes precipitated with protein A/G beads. As shown in Fig. 7B, some MHC class I molecules were co-precipitated by anti-HA mAb (sample 4, upper blot), and some BILF1 molecules were co-precipitated by W6/32 mAb (sample 2, lower blot). In the control TfR immunoprecipitations, no co-precipitation of MHC class I or BILF1 was observed (sample 6).

We next asked whether BILF1 enhances the endocytosis of surface MHC molecules. In MJS cells, BILF1 substantially increased the rate of disappearance of MHC class I molecules from the cell surface, but did not affect the rate of disappearance of MHC class II molecules (Fig. 7C). Similar results were obtained with LCLs that had been transduced with a BILF1 retrovirus (data not shown). In 293 cells, expression of BILF1 reproducibly increased the rate of disappearance of MHC class I molecules from the cell surface, although the magnitude of the effect was masked by the unusually high rate of disappearance in the control 293 cells (Fig. 7D, upper graph). Importantly, the rate of appearance of new MHC class molecules at the cell surface was identical for the first 30 min of analysis; thereafter, the appearance of new MHC class I molecules reached a plateau in BILF1^+^ cells, while new molecules continued to accumulate for at least a further 30 min in control 293 cells (Fig. 7D, lower graph). We interpret the differences between control and BILF1 cells in these latter experiments to be due to the effect of the increased rate of internalisation of MHC class I molecules that becomes apparent after 20–30 min (see Fig. 7D, upper graph). Together, the results in Fig. 7A–D suggest that BILF1 targets the mature MHC class I complexes for degradation after they reach the cell surface.

### Lysosomal inhibitors block BILF1-induced degradation of MHC class I

**Figure 8:**
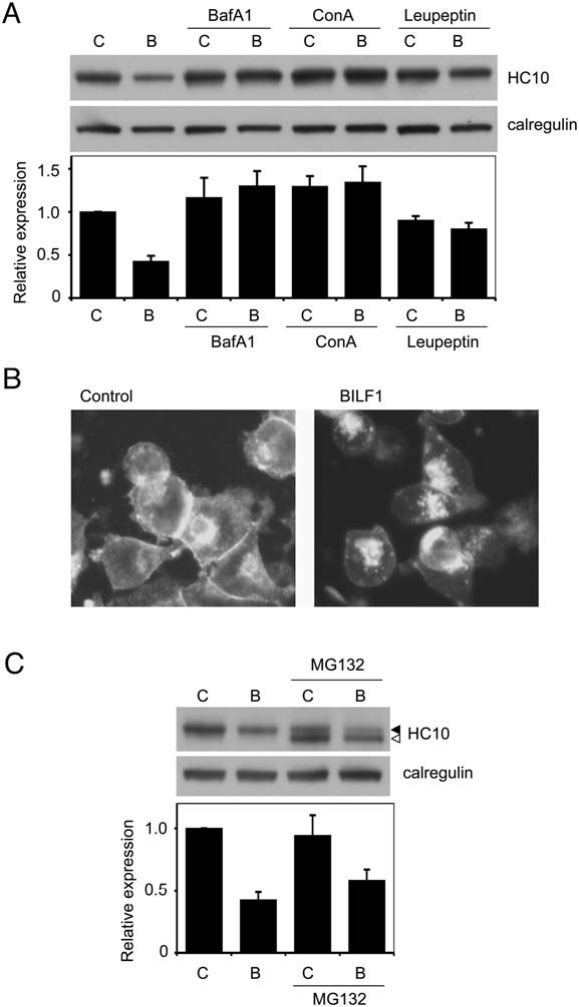
Lysosomal inhibitors block BILF1-enhanced degradation of MHC class I. (A) 293 cells stably transduced with control- (c) or BILF1- (b) retrovirus were treated with or without Bafilomycin A1, concanamycin A, or leupeptin for 20 hr. Lysates from 2×10^5^ cell equivalents were separated by SDS/PAGE gel, and analyzed by western blotting using antibodies specific for MHC class I (HC10) and calregulin. The blot is one representative of three independent experiments. The histogram shows the mean results (±S.D.) of quantification by densitometry of all the blots from 3 independent experiments, where the densities of the HC10 bands were normalized relative to their own calregulin loading control. (B) 293 cells stably transduced with control or BILF1 retrovirus were treated with concanamycin A (50 nM) for 6 hr prior to fixation and permeabilization with methanol/acetone, then stained with W6/32 primary antibodies and Alexa Fluor® 488 goat anti-mouse IgG secondary antibodies. The photographs were obtained with a conventional fluorescence microscope. (C) 293 cells stably transduced with control (c) or BILF1 (b) retrovirus were treated with or without the proteasome inhibitor, MG132, for 20 hr, and analyzed by western blot as in panel A. The additional, lower molecular weight species detected is probably deglycosylated and/or partially degraded free heavy chain that is normally targeted for proteasomal degradation.

We used a panel of inhibitors to identify the mechanism of BILF1-induced degradation of MHC class I molecules, including: bafilomycin A1 and concanamycin A, which can inhibit proteolysis by raising endolysosomal pH [30],[31]; leupeptin, which inhibits cysteine and serine proteases [32]; and the proteasome inhibitor, MG132 [33]. Control and BILF1^+^ 293 cells were incubated with or without these inhibitors for 20 hr before harvesting the cells for Western blot analysis. The representative blot probed with HC10 in Fig. 8A shows that bafilomycin A1, concanamycin A, and leupeptin all restored the level of MHC class I heavy chains in BILF1-293 cells to the same levels seen in control-293. The blots from three independent experiments were quantitated by densitometry, and the mean values shown in the histogram beneath the blot in Fig. 8A.

Fluorescence microscopy of BILF1^+^ cells treated with lysosomal inhibitors revealed a marked accumulation of intracellular MHC class I staining that was not observed in BILF1^−^ cells treated with lysosomal inhibitor (Fig. 8B), nor in BILF1^+^ cells treated with the proteasome inhibitor, MG132 (data not shown). Furthermore, quantitation of the total cell MHC class I protein in Western blots revealed that the BILF1^+^ 293 cells retained their overall reduced expression of MHC class I molecules relative to control 293 cells following treatment with the proteasome inhibitor (Fig. 8C).

Taken together, the results in Figs. 7 and 8 suggest that the accelerated degradation induced by BILF1 occurs within an endolysosomal compartment following internalisation of MHC class I complexes from the cell surface.

### Comparison of BILF1/GPCR homologs

**Figure 9:**
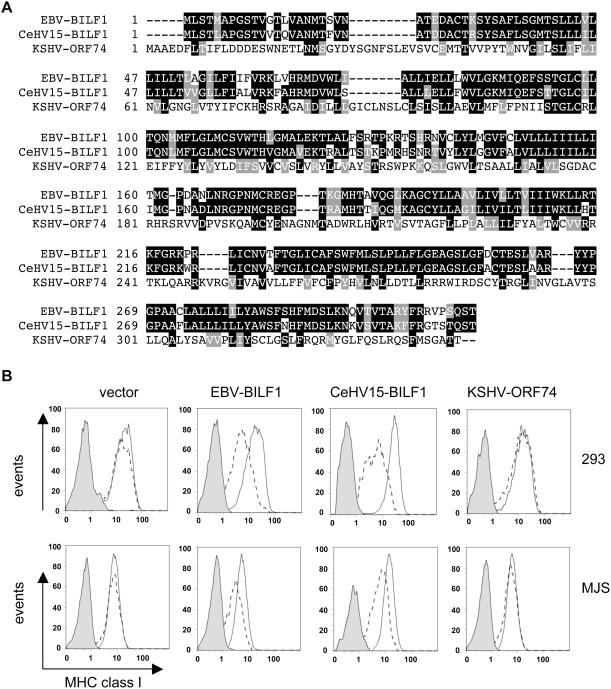
The ability of BILF1 homologs to downregulate MHC class I. (A) Multiple sequence alignment of EBV-BILF1, the rhesus lymphocryptovirus CeHV15-BILF1, and KSHV-ORF74. The alignment was done with ClustalW version 1.8, and shading was done with Boxshade version 3.21, available at http://www.ch.embnet.org/software/BOX_form.html. (B) 293 or MJS cells were transfected with EBV-BILF1, CeHV15-BILF1 or KSHV-ORF74 genes in the bicistronic vector, pCDNA3-IRES-nlsGFP. At 48 hr post-transfection, surface MHC class I molecules were stained and analyzed exactly as in Fig. 1.

It is well established that many, if not all, herpesviruses encode proteins with features of GPCRs [27]. The most closely related vGPCR to BILF1 amongst the human herpesviruses is the ORF74 product of KSHV (a γ_2_-herpesvirus), which shares just 15% amino acid sequence identity and 29.7% similarity with BILF1 (Fig. 9A). However, a more closely related herpesvirus , CeHV15 (a γ_1_-herpesvirus, as is EBV), of the Rhesus Old World primate encodes a BILF1 homolog sharing 80.4% amino acid sequence identity and 88.5% similarity with EBV BILF1 (Fig. 9A).

In order to investigate whether these vGPCRs shared with EBV BILF1 the ability to downregulate MHC class I, we cloned them into the bicistronic vector pCDNA3-IRES-nls-GFP and transiently transfected them into 293 and MJS cells, and analysed their effect on cell surface MHC class I expression exactly as in Fig. 1. The results illustrated by a representative experiment in Fig. 9B, show that Rhesus CeHV15-BILF1 clearly did reduce MHC class I expression at the cell surface, but that KSHV-ORF74 did not.

### Downregulation of MHC class I by BILF1 is independent of its signaling function

**Figure 10:**
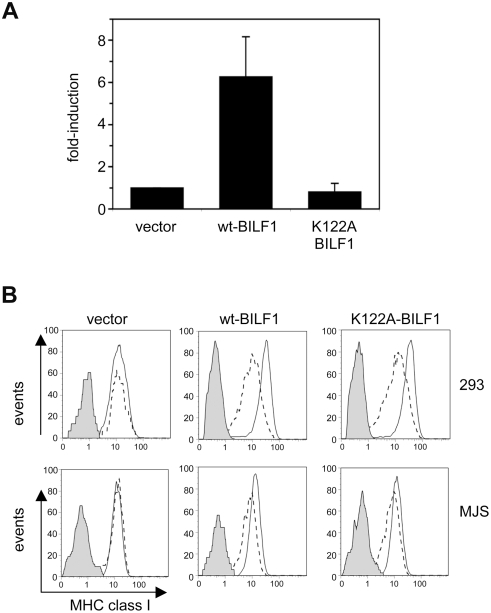
BILF1 signaling function is not required to downregulate MHC class I. (A) Wild-type or K122A-mutant BILF1 expression plasmids were transfected into the HEK293-NFκB reporter cell line, and the degree of NFκB activation measured by detection of luciferase activity. The results are the mean±S.D. for three independent experiments performed in triplicate. (B) 293 and MJS cells were transfected with wild type BILF1 or mutant K122A-BILF1 genes in the bicistronic vector, pCDNA3-IRES-nlsGFP. At 48 hr post-transfection, surface MHC class I molecules were stained and analyzed exactly as in Fig. 1.

Since different vGPCRs activate different G-proteins and signalling pathways, the results in Fig. 9 could be interpreted as indicating that shared signalling functions of the EBV and CeHV15 vGPCRs determine their ability to downregulate MHC class I. To test this hypothesis, we obtained a signaling-negative mutant of EBV BILF1. The BILF1 polypeptide contains an EKT sequence in its predicted third transmembrane domain, which represents a conservative amino acid substitution of the ‘DRY box’ (i.e. residues with acidic, basic, polar side chains), known to be important in G-protein signaling [27],[34]. Substitution of an alanine for lysine at residue 122 abolished G-protein activation and BILF1 signaling function, as we observed by transfecting wild-type BILF1 or the K122A-BILF1 mutant into the HEK293-NFκB reporter line. The results in Fig. 10A are the mean of three independent experiments, and show that whereas transfection of wt-BILF1 typically induced more than a 5-fold increase in NFκB activity relative to the empty vector control transfectant, the K122A-BILF1 mutant did not induce any discernable NFκB activation above the vector control. In both 293 and MJS cells, transient transfection of the mutant K122A-BILF1 was able to cause downregulation of MHC class I (Fig. 10B). These results suggest that the ability of BILF1 to target MHC class I molecules for endolysosomal degradation is not critically dependent upon its signaling function.

## Discussion

In this study we have shown that the BILF1 protein, which was recently identified as a constitutive signaling vGPCR [25],[26], can downregulate cell surface MHC class I and abrogate EBV-specific T cell recognition. The essential elements of the mechanism of this immune evasion function have been elucidated. Pulse–labeling and immunoprecipitation experiments with BILF1*-*transduced cells revealed that BILF1 associates with MHC class I molecules within 15 mins of biosynthesis. The MHC class I molecules can mature to the Endo H-resistant glycosylated form as normal, but were more rapidly internalized from the cell surface and were degraded by lysosomal enzymes. The effects of BILF1 on MHC class I were not critically dependent upon the activation of G-protein signaling, and were not exhibited by the most closely related human herpesvirus GPCR homolog, the ORF74 protein of KSHV, which has 15% amino acid sequence identity with EBV BILF1. Interestingly, the BILF1 homolog of a Rhesus γ_1_-herpesvirus (CeHV15 BILF1, showing 80% amino acid identity with EBV BILF1) did downregulate MHC class I similarly to EBV BILF1. This observation reinforces the degree of similarity in the sequences and functions of the EBV and CeHV15, and strengthens the case for CeHV15 infection of Rhesus Macaques being an excellent animal model for understanding the interplay between immune responses and virus-infected cells during persistence of the virus in the healthy host and the development of virus-induced tumors [23],[35],[36].

Our *in vitro* studies, identifying a new function for BILF1, add to the complexity of the immune-evasion mechanisms of Epstein-Barr virus during lytic replication. EBV has now been demonstrated to target the MHC class I antigen processing pathway at the level of translation, through BGLF5 [22],[24]; peptide transport, through BNLF2a [23]; and by targeting mature MHC class I molecules for degradation, through BILF1. BGLF5 and BNLF2a are both products of early lytic cycle genes [24],[37], but there are conflicting reports on the early/late status of *BILF1* [25],[26],[37]. Our own results (D. van Leeuwen, unpublished) show that induction of BILF1 mRNA expression during lytic cycle is insensitive to phosphonoacetic acid treatment, indicating that it is an early gene, although its appearance is delayed relative to BNLF2a transcripts. The temporal expression of BNLF2a, BGLF5 and BILF1 proteins and their impact at different points along the antigen processing pathway, suggest that their cooperative effects will cause efficient abrogation of antigen presentation, particularly at the later stage of lytic cycle. This is consistent with the observation that CD8^+^ T cell responses to late lytic cycle antigens are rare compared to the responses to immediate-early and early antigens [20]. It has also been suggested that the viral IL-10 homolog (vIL-10) produced by the *BCRF1* gene might be involved in evasion from CD8^+^ T cell responses since vIL-10 can selectively reduce levels of TAP-1 mRNA, and therefore peptide transport, in human B cells [38]. However, since *BCRF1* is a late gene, any autocrine effects of vIL-10 on TAP-1 mRNA would be masked by the earlier expression of the host-shutoff protein, BGLF5 [22],[24]. In the context of immune-evasion, it is likely that secreted vIL-10 may be more relevant to bystander B cells being infected by the virus released from cells undergoing lytic replication *in vivo*, coupled with the effects of vIL-10 on T cell and NK cell functions.

BILF1 is a seven transmembrane segment GPCR which, like many vGPCRs encoded by herpesviruses, shares structural and functional characteristics with chemokine receptors [25]–[27]. One role of viral chemoreceptors is to contribute to efficient lytic replication cycle by reprogramming the host cell through multiple signaling pathways [27], a process that might aberrantly lead to cellular transformation and oncogenesis, as has been suggested for KSHV ORF74 [39]–[41]. In addition, it has long been recognized that viral chemoreceptors have the potential to contribute to immune-evasion strategies. For example, Bodaghi and colleagues reported in 1998 that one of the four vGPCRs of HCMV, encoded by *US28*, can modify the chemokine environment of infected cells by binding and internalizing MCP-1 (monocyte chemoattractant protein) and RANTES (regulated on activation, normal T cell expressed and secreted) to efficiently sequester these chemokines. In certain cellular contexts, i.e. latent infection of monocytes with HCMV, US28 can also transcriptionally upregulate MCP-1 expression [42], whilst during lytic cycle in fibroblasts the transcription of MCP-1 is downregulated [43], though not by US28. Similarly to HCMV US28, the U51 GPCR of HHV6 is also able to bind and sequester RANTES and MCP-1, but the HHV6 U51 additionally induces transcriptional downregulation of RANTES [44]. These examples of transcriptional regulation of immune-modulating chemokines by vGPCRs of β-herpesvirus are likely to be more significant in the lytic cycle of this virus subfamily, where host protein synthesis shutoff is not a feature of lytic cycle as it is in α- and γ-herpesviruses [22].

Our observation with BILF1 represents the first example of a viral GPCR/chemokine receptor interfering directly with the MHC class I antigen processing pathway. Interestingly, a similar phenomenon was recently reported for the cellular chemokine receptor, CXCR4 [45]. Thus, CXCL12 ligand-induced activation of CXCR4 on malignant cancer cell lines and peripheral blood mononuclear cells triggered down-regulation of MHC class I from the cell surface. Mechanistically, this process involves physical association of CXCR4 with the β2-microglobulin component of mature MHC class I complexes, ubiquitination of the MHC class I heavy chain, and endocytosis of MHC complexes into a late endosomal/lysosomal compartment where degradation presumably occurs. Although BILF1 is unable to directly associate with β2-microglobulin (JZ, unpublished data) and we have no evidence that BILF1 induces ubiquination of MHC class I heavy chains above the levels seen in control BILF1^−^ cells (Fig. 6), CXCR4 does share some broad features with the mechanism by which BILF1 downregulates MHC class I, which raises the possibility that targeting of the antigen processing pathway might not be a property uniquely associated with the γ_2_-herpesvirus BILF1 vGPCRs. It is perhaps relevant that when co-expressed in the same cell, BILF1 and CXCR4 show almost complete co-localization (both being present predominantly in the plasma-membrane), whereas the KSHV-ORF74 and HCMV-US28 vGPCRs (predominantly intracellular) exhibit separate localizations to BILF1 [25].

Many other viral gene-products have been shown to target MHC class I molecules for degradation. Some, such as the US3, US10, US2 and US11 proteins of HCMV, target MHC molecules before they reach the cell surface, either by interfering with the maturation of MHC class I molecules and their egress from the ER [5]–[8] or by inducing retrograde transport from the ER to the cytosol with subsequent degradation by proteasomes [9]–[11]. Other viral proteins, such as the KSHV K3 and K5 proteins [29],[46],[47], induce endocytosis and lysosomal degradation of cell surface MHC class I complexes. Although BILF1 shares no obvious structural or sequence properties with any of these viral proteins, the mechanism of interference with MHC class I by BILF1 shares many features with those proteins that induce endocytosis of cell surface MHC class I molecules and subsequent degradation. However, whereas, KSHV K3 and K5 proteins were shown to induce ubiquitination of MHC class I heavy chains, which appears to be a signal for endocytosis and subsequent degradation [48],[49], we found no evidence that BILF1 induced ubiquitination of MHC class I heavy chains above the level seen in control BILF1-negative cells. Furthermore, BILF1 lacks a plant homeodomain (PHD) or RING-finger motif present in K3 and K5 proteins that are necessary for their ability to induce ubiquitination of MHC class I molecules [49]–[51].

Some viral proteins, such as murine CMV gp48 [28] and the HHV-6A/B U21 proteins [52], contain dileucine motifs in their cytosolic C-terminus that have been shown to be important for lysosomal targeting of MHC class I complexes, but no dileucine motif is present in the cytosolic domains of BILF1. The HIV-1 Nef protein contains a typical di-leucine motif that is involved in the binding of adapter proteins involved in membrane trafficking [53],[54], although mutation of this motif does not affect the ability of Nef to downregulate MHC class I [55]. Indeed, although Nef was one of the first viral proteins reported to induce endocytosis and degradation of MHC class I molecules from the cell surface [56], it is now unclear to what extent endocytosis of MHC class I accounts for its downregulation by Nef. An alternative explanation is that Nef acts predominantly by diverting intracellular MHC class I complexes to endosomes and lysosomes before they can reach the plasma-membrane [57]–[59]. This would make Nef more similar to murine CMV gp48 which also diverts MHC class I from the ER to the lysosomal compartment for degradation [28].

Since BILF1 is predominantly located in the plasma membrane, our data on BILF1 suggest that endocytosis of surface MHC class I molecules associated with BILF1 is likely to be the main mechanism of targeting the MHC class I molecules to the lysosome. However, we cannot yet rule out the possibility that some MHC class I molecules might also be diverted before they reach the surface. An investigation of what adaptor molecules can associate with BILF1 will provide crucial information to elucidate the finer details of BILF1's mechanism of action. It also remains to be determined what are the sequence elements of BILF1 that are involved in its association with MHC class I molecules. Experiments are ongoing to resolve these unanswered questions.

It is perhaps surprising that BILF1 can inhibit T cell recognition so efficiently when it has a comparatively small effect on the levels of surface MHC class I (see Figs. 2–4). There are a number of possible explanations for this. First, BILF1 might target different HLA alleles with different efficiency. Thus, while HLA-A2, -B8, and B35 might be efficiently removed from the cell surface for degradation, other alleles recognized by W6/32 antibody may be unaffected and will mask the full extent that selected MHC molecules are being degraded. Secondly, the association of BILF1 with MHC class I molecules in the ER might interfere with Tapasin or TAP binding and thus interfere with correct peptide loading, while stabilizing a defective MHC complex and allowing egress from the ER. Thirdly, the association of BILF1 with MHC class I molecules at the cell surface might directly interfere with recognition by TCR. None of these possibilities is mutually exclusive, and further work is ongoing to test them.

In summary, through identifying and partially characterizing the molecular mechanisms of an immune-evasion function for BILF1, we have extended our understanding of the normal biology of an important human pathogen. The discovery of a third EBV lytic cycle gene that cooperates to interfere with MHC class I antigen processing underscores the importance of the need for EBV to be able to evade CD8^+^ T cell responses during the lytic replication cycle, at a time when such a large number of potential viral targets are expressed.

## Materials and Methods

### Plasmids and retroviral expression vectors

The EBV lytic genes *BMRF2*, *BILF1*, *BILF2*, *and BXLF2* were PCR-amplified from the B95.8 virus sequences, and *LF1* and *LF2* from Raji EBV sequences, within the Bacterial artificial Chromosome (BAC) plasmid, 2089 [60]. The KSHV gene *ORF74* was PCR-amplified from a KSHV BAC [61]. The *BILF1* homolog of a Rhesus lymphocryptovirus was PCR-amplified from a cosmid derived from the CeHV15 virus, [62] that was kindly provided by F. Wang (Harvard Medical School, Boston). All the viral genes were subcloned into the EcoRI/NotI sites of pCDNA3-IRES-nls-GFP vector with an additional 5′-HA tag. All plasmids were verified by restriction digest and sequence analysis. EBV *BNLF2a* with 3′-HA tag in pCDNA3-IRES-nls-GFP vector has been described previously [23]. Expression plasmids p509, containing the EBV *BZLF1* gene, and pCEP4-SM, containing the EBV *BSLF2/BMLF1* spliced gene, have been described previously [24].

Two retroviral vector systems were used. The Retro-X™ Universal Packaging System (Clontech Laboratories, Inc.) was used to prepare retrovirus based on the PQCXIH vector for hygromycin drug selection. The alternative vector, pLZRS-IRES-GFP, instead allowed coexpression of the marker gene GFP. The BILF1 gene with a 5′-HA tag was subcloned into each vector.

### Cells and transfections

The HEK293 epithelial cell line (American Type Culture Collection) and derived cell lines were maintained in Dulbecco's modified Eagle's medium (DMEM) supplemented with 10% fetal bovine serum and penicillin-streptomycin antibiotics. The HEK293-NFκB-luc cell line (kindly provided by G. Lipford, Munich) contained a stably transfected NFκB-luciferase promoter-reporter gene [63], and was maintained in medium supplemented with 0.7 mg/ml G418 as a selective agent. The MJS (Mel JuSol) melanoma-derived cell line [64] was maintained in RPMI-1640 medium supplemented with 10% fetal bovine serum and penicillin-streptomycin antibiotics. 293 and MJS cell lines transduced by PQCXIH-based retroviruses were maintained in their medium with 400 µg/ml hygromycin. For some experiments, 293 and MJS cells were transiently transfected with plasmid DNA using Lipofectamine 2000 (Invitrogen). EBV specific CD8^+^ cytotoxic T cells were grown in 10% FCS in RPMI-1640 medium supplemented with 30% supernatant from the IL-2-producing MLA 144 cell line [65] and 50 U/ml recombinant IL-2 as described elsewhere [20].

### Retroviral infection

For the transduction of 293 cells and MJS cells, the packaging cell line (GP2-293) was transfected with retroviral vectors (PQCXIH or PQCXIH-BILF1) and PVSV-G to produce replication-defective viral particles. The supernatants containing recombinant retrovirus were harvested 48 hours after transfection, and filtered through a 0.22-µm pore, low protein-binding filter. To generate stable cell lines by transduction with PQCXIH-BILF1 or control PQCXIH-BILF1 retrovirus, 293 cells or MJS cells were infected with 1 ml retrovirus supernatants with polybrene added to 4 µg/ml final dilution. The stable cell lines were selected by 400 µg/ml hygromycin.

For retrovirus transduction of LCLs, the packaging cell line (GP2-293) was transfected with retroviral vectors (PLZRS-IRES-GFP or PLZRS-BILF1-IRES-GFP) and PVSV-G to produce replication-defective viral particles. To generate stable cell lines by transduction with these viral vectors, the LCLs were infected with retrovirus supernatants and the GFP^+^ cells were sorted on a Cytomation MoFlo cell sorter at 72 hr post-infection.

### Antibodies

The BZ.1 murine mAb specific for the EBV BZLF1 encoded protein was generated in the authors' laboratory [66]. Murine mAbs used to detect human MHC class I were: W6/32 [67] which recognizes native β2m-associated MHC class I (HLA-A, -B, and -C alleles) complexes; and HC10 [68], recognizing free HLA class I heavy chains. The DA6.147 murine mAb specific for HLA-DR α-chains was obtained from the ATCC, and mAb L234 [69] specific for HLA-DR β-chains, was kindly provided by P. Cresswell (Howard Hughes Medical Institute, New Haven). For flow cytometry experiments, antibodies for detecting MHC class I and class II were purchased from Serotec, including phycoerythrin(PE)-conjugated anti-HLA A,B,C (MCA81PE; clone W6/32) andPE-conjugated anti-HLA DR (MCA71PE; clone YE2/36-HLK). The murine mAbs specific for the TAP1, 148.3 [70], and TAP2, 435.3, were kindly provided by R. Tampe (Wolfgang Goethe-University, Frankfurt) and by P.M. van Endert (Institute Necker, Paris) respectively. The murine mAb, H68.4, to human transferrin receptor (TfR) protein was purchased from Roche Diagnostics, and Goat antibodies to calregulin (sc6467) were purchased from Santa Cruz Biotechnology. Rat mAb, 3F10, and mouse mAb, 12CA5, directed against the influenza virus derived HA tag, were purchased from Roche Diagnostics and Santa Cruz Biotechnology respectively.

### Flow cytometry analysis of cell surface MHC molecules

Cell surface expression of MHC class I on viable cells was determined by staining with PE-labeled W6/32 antibodies or PE-labeled isotype control mAb (both from Serotec) and detection on a Beckman Coulter XL flow cytometer. The data were analysed using Flowjo software (Tree Star).

To assay the kinetics of internalization of surface MHC class I and class II molecules, 293 cells or MJS cells were incubated for 60 min on ice with saturating amounts of W6/32 or L234 mAb, then washed three times in phosphate-buffered normal saline (PBS) and replaced in warm culture medium and incubated at 37°C for the length of time indicated in the results section. To terminate MHC/antibody complex internalization, cells were rapidly cooled to 0°C. Finally, the mAb-bound surface MHC molecules were stained at 0°C with PE-conjugated goat anti-mouse IgG2a antibody (Serotec), and cells were analyzed by flow cytometry.

To assay the kinetics of appearance of surface MHC class I, 293 cells were incubated for 60 min on ice with saturating amounts of W6/32, then washed three times in PBS, replaced in warm culture medium and incubated at 37°C for the length of time indicated in the results section. To terminate further appearance of new surface MHC-I, cells were rapidly cooled to 0°C. Finally, the newly appeared surface MHC-I molecules were stained with PE-conjugated W6/32, and the cells were analyzed by flow cytometry.

### Western Blots

Total cell lysates were denatured in reducing sample buffer (final concentration: 2% SDS, 72.5 mM Tris-HCl pH 6.8, 10% glycerol, 0.2 M sodium 2-mercaptoethane-sulfonate, 0.002% bromophenol blue), then sonicated and heated to 100°C for 5 minutes. Solubilized proteins equivalent to 2×10^5^ cells/20 µl sample were separated by sodium dodecyl sulfate/polyacrylamide gel electrophoresis (SDS/PAGE) on 4–12% gradient Bis-Tris NuPage mini-gels with MOPS running buffer (Invitrogen). Following electroblotting to polyvinylidene difluoride membranes (Invitrogen) and blocking with I-Block (Tropix, Applied Biosystems) in phosphate-buffered saline and 0.1% Tween-20 detergent, specific proteins were detected by incubating the membranes with primary antibodies at 4°C overnight. The 148.3 and 435.3 mouse anti-TAP1 and TAP2 mAbs were used at 1/100 dilution of culture supernatant, whilst the DA6.147 anti-HLA-DR was used at a 1/250 dilution; the purified mouse mAbs, BZ.1, H68.4, 12CA5, HC10, were used at 1 µg/ml; the rat anti-HA mAb was used at 50 ng/ml; and the goat antibody to calregulin, was used at 1 µg/ml. Primary antibodies specifically bound to blotted proteins were detected by incubation for 30 min with appropriate alkaline phosphatase conjugated secondary antibodies, then were developed using a CDP-Star™ detection kit (Tropix, Applied Biosystems) and exposed to autoradiographic film. In one set of experiments, the alkaline-phosphatase-conjugated CleanBlot™ immunoprecipitation dection reagent (Thermo Scientific) was used at a 1/500 for 60 min to detect blots of immunoprecipitates being probed with the HC10 mAb.

### T cell function assays

The effector T cell clone, RAK, specific for BZLF1 and restricted through HLA-B8, was generated as described elsewhere [20]. Targets for the RAK clone were generated by transfection of MJS cells (HLA-B8 type) with a BZLF1 expression plasmid with or without a BILF1 expression plasmid. At 24 hr post-transfection, recognition of target cells by the effector T cells was determined by ELISA of IFN- γ release using a standard protocol described elsewhere [71]. Briefly, 10^4^ effector T cells were incubated for 18 hr at 37°C in V-bottom microtest plate wells with 10^5^ target cells, then the supernatants were harvested for quantitation of IFN-γ by ELISA (Endogen) in accordance with the manufacturer's recommended protocol. Specificity control targets included HLA-matched and HLA-mismatched EBV-transformed lymphoblastoid cell lines (LCL), empty vector-transfected MJS cells, and empty vector-transfected MJS cells pulsed with the RAKFKQLL synthetic peptide.

LCLs transformed with a BZLF1^−^ recombinant EBV (to prevent induction of endogenous lytic cycle genes) were transduced with control PLZRS-IRES-GFP or PLZRS-BILF1-IRES-GFP retroviruses. These lines were used as targets in assays with a panel of effector T cell clones specific for constitutively expressed latent EBV genes, EBNA1, EBNA3A, LMP2 or BHRF1. The effector T cell clone, HPV, was a CD8^+^ line specific for EBNA1 and restricted through HLA-B35 [72]; clone YPL was a CD8^+^ line specific for EBNA3A and restricted through HLA-B35 [72]; clone CLG was a CD8^+^ line specific for LMP2A and restricted through HLA-A2 [73]; clone PYY was a CD4^+^ line specific for BHRF1 and restricted through DR4 (Kelly, G.L., Long, H.M. et al, submitted for publication). Recognition of target cells by the effector T cells was determined by ELISA of IFN- γ release using a standard protocol described elsewhere [71].

### Metabolic Labeling, Immunoprecipitation, and Endo H Digestion

Cells (10^7^) were starved with 15 ml methionine-free DMEM medium supplemented with 10% dialysed FCS for 1 h at 37°C, then labeled for 15 min with 200 µCi of EasyTag Express ^35^S protein labeling mix (NEN Life Science Products) in a final volume of 1 ml. After two washes with chase medium (normal DMEM medium supplemented with 10% FCS), the cells were resuspended at 2×10^6^ cells/ml and chased at 37°C for the times indicated. Samples containing 2×10^6^ cells were lysed in 400 µl of NP-40 buffer (0.5% Nonidet P-40, 5 mM MgCl_2_ and 50 mM Tris-HCl, pH 7.5) with protease inhibitor cocktail (Sigma) at 4°C for 45 min. Nuclei and insoluble debris were removed by centrifugation, and the supernatants were precleared first with 1.2 µl of normal mouse serum and 20 µl Dynabeads Protein A (Invitrogen) for 2 hr at 4°C, and then again with 20 µl Dynabeads Protein A and 20 µl Dynabeads protein G at 4°C overnight. The precleared lysates were immunoprecipitated for 2 hr with 6 µl of W6/32 Miniperm™ culture supernatant and 20 µl Dynabeads Protein A plus 20 µl Dynabeads protein G, before washing the beads four times with NET buffer (0.5% NP-40, 150 mM NaCl_2_, 5 mM EDTA and 50 mM Tris-HCl, pH 7.5) and eluting by boiling in reducing gel sample buffer for 5 min. For endoglycosidase H (Endo H) treatment, 20 µl samples were incubated at 37°C for 2.5 hr with 2 µl G5 reaction buffer and 1.5 µl of Endo H enzyme (New England Biolabs Inc.). For second-round precipitations, boiled eluates were first diluted 10-fold with lysis buffer, then reprecipitated as above with either HC10 (MHC class I heavy chain) or 3F10 (anti-HA tag on BILF1). Finally, the samples were separated by SDS-PAGE on 10% Bis-Tris NuPage mini-gels with MOPS buffer (Invitrogen). After the gels were fixed and dried, they were exposed to autoradiographic film.

### Cold co-immunoprecipitations

For immunoprecipitations from whole cell lysates, 2×10^6^ cells were lysed in 400 µl of NP-40 buffer with protease inhibitors and were immunoprecipitated as above. Lysates were precipitated with 1 µg of either W6/32 (HLA-ABC), 12CA5 (HA-tag; BILF1) or H68.4 (TfR) mAbs. Eluted samples were separated on 4–12% Bis-Tris NuPage mini-gels with MOPS buffer, electroblotted to polyvinylidene difluoride membranes and probed with antibodies HC10 (anti- HLA class I heavy chain), H68.4 (anti-TfR) or 3F10 (anti-HA tag on BILF1). For the selective immunoprecipitation of cell-surface surface molecules, 2×10^6^ cells were incubated for 60 min on ice with saturating amounts of W6/32, H68.4 or 12CA5, then washed three times in PBS. The cells were then lysed in 400 µl of NP-40 buffer with protease inhibitors and immunoprecipitated with 20 µl Dynabeads Protein A plus 20 µl Dynabeads protein G.

### Immunofluorescence staining of fixed cells

293-BILF1 cells were grown on glass slides coated with fibronectin, and were treated with concanamycin A for 6 hrs. For the confocal microscopy, the cells were rinsed with PBS and fixed with 4% (w/v) paraformaldehyde in PBS for 20 min, then permeabilized with 0.5% Triton X-100 in PBS for 5 min. For conventional fluorescence microscopy, the cells were fixed and permeabilized with methanol-acetone (1∶1 vol∶vol) for 15 min at −20°C. The slides were incubated with primary antibodies for 2 hr at 37°C, washed extensively in PBS, then incubated with Alexa Fluor® 488 goat anti-mouse IgG or Alexa Fluor® 594 goat anti-rat IgG secondary antibodies (Invitrogen) for 45 min. After repeated washing the slides were mounted with vectashield mounting medium with DAPI stain (Vector laboratories) and analyzed with a Zies LSM510 laser scanning confocal microscope, or with a Nikon E600 conventional fluorescence microscope.

### Protease inhibitor assays

293 cells stably transduced with control or BILF1 retrovirus were treated with Bafilomycin A1 (2 µM), concanamycin A (50 nM), or leupeptin (200 µM) or MG132 (10 µM) for 20 hr. All inhibitors were purchased from Sigma-Aldrich. Lysates from 2×10^5^ cell equivalents were separated by SDS/PAGE gel, and analyzed by Western Blotting using antibodies specific for MHC class I (HC10) and calregulin.

### NFκB reporter assays

HEK293-NFκB-luc cells were seeded at 2×10^5^/ml in a 96-well plate at 24 hr prior to transfection with a constitutively expressed *Renilla*-luciferase reporter construct (phRL-TK, 70 ng/well, Promega) for normalizing transfection efficiency, together with BILF-wild-type, BILF1-K122A mutant, or empty vector DNA (160 ng/well). The Lipofectamine-2000 (Invitrogen) and DNA mix in Opti-MEM medium was prepared according to the supplier's instructions, and 50 µl was then added to each well. Cell extracts were generated after 48 h using Cell Culture Lysis Buffer (Promega), and extracts were assayed for firefly luciferase and *Renilla*-luciferase activity using the Luciferase Assay System and *Renilla* Luciferase Assay System (Promega), respectively.
